# Gait Rather Than Cognition Predicts Decline in Specific Cognitive Domains in Early Parkinson’s Disease

**DOI:** 10.1093/gerona/glx071

**Published:** 2017-05-03

**Authors:** Rosie Morris, Sue Lord, Rachael A Lawson, Shirley Coleman, Brook Galna, Gordon W Duncan, Tien K Khoo, Alison J Yarnall, David J Burn, Lynn Rochester

**Affiliations:** 1 Institute of Neuroscience; 2 Newcastle Institute for Ageing; 3 UK and Industrial Statistics Research Unit; 4 School of Biomedical Sciences, Newcastle University, Newcastle upon Tyne, UK.; 5 Centre for Clinical Brain Science, University of Edinburgh, UK.; 6 School of Medicine and Menzies Health Institute Queensland, Griffith University, Australia.; 7 School of Medicine, University of Wollongong, New South Wales, Australia

**Keywords:** Biomarker, Dementia, Gait

## Abstract

**Background:**

Dementia is significant in Parkinson’s disease (PD) with personal and socioeconomic impact. Early identification of risk is of upmost importance to optimize management. Gait precedes and predicts cognitive decline and dementia in older adults. We aimed to evaluate gait characteristics as predictors of cognitive decline in newly diagnosed PD.

**Methods:**

One hundred and nineteen participants recruited at diagnosis were assessed at baseline, 18 and 36 months. Baseline gait was characterized by variables that mapped to five domains: pace, rhythm, variability, asymmetry, and postural control. Cognitive assessment included attention, fluctuating attention, executive function, visual memory, and visuospatial function. Mixed-effects models tested independent gait predictors of cognitive decline.

**Results:**

Gait characteristics of pace, variability, and postural control predicted decline in fluctuating attention and visual memory, whereas baseline neuropsychological assessment performance did not predict decline.

**Conclusions:**

This provides novel evidence for gait as a clinical biomarker for PD cognitive decline in early disease.

Cognitive impairment is significant in Parkinson’s disease (PD) (1) with progression to PD dementia (PDD) highly prevalent in advanced disease (2). It significantly impacts on daily functioning and quality of life (3), and ultimately reduces life expectancy (4). Detecting “at risk” individuals in early disease is of upmost importance to optimize clinical management and progress novel therapeutics. However, clinical biomarkers continue to be sought to address this unmet need.

Cognitive decline in PD is complex and the underlying pathophysiology poorly understood. Deficits arise predominantly from dopaminergic and cholinergic dysfunction (5) which interact and impact selectively on different cognitive functions, yielding heterogeneous cognitive patient profiles (1). Because of this complexity, a single biomarker to predict cognitive decline and dementia in PD is unlikely to be sufficient; therefore, a combinatorial approach is considered optimal (5). Clinical biomarkers make an important contribution to a combinatorial battery given the complexity, cost, and invasive nature of some laboratory or imaging biomarkers (5).

Gait has potential as a low-cost and noninvasive clinical biomarker for cognitive decline and dementia in PD based on findings which show gait changes precede and predict cognitive decline and dementia in ageing (6–8). Shared neurochemical and pathological mechanisms of gait and cognition explain this relationship and support the potential of gait characteristics as discrete clinical biomarkers of cognitive decline and dementia. Although there is a robust relationship between gait and cognition in early PD, a comprehensive approach to the longitudinal nature of the relationship has yet to be established (9,10). Moreover, previous work lacks a consistent and detailed approach to evaluating gait characteristics, limiting interpretation (10,11).

This study utilized a comprehensive battery of gait and cognitive characteristics to determine (i) if gait can predict cognitive decline in early PD, (ii) if gait characteristics are global or specific predictors, and (iii) if gait is more sensitive than cognition in predicting cognitive decline. Based on current literature (10) and our previous cross-sectional work (9), we hypothesized that discrete gait characteristics will be sensitive to cognitive decline in early PD.

## Methods

### Participants

Subjects with newly diagnosed idiopathic PD were recruited to ICICLE-Gait, a nested study within Incidence of Cognitive Impairment in Cohorts with Longitudinal Evaluation-PD (ICICLE-PD) (12). Potential participants were recruited between June 2009 and December 2011. Idiopathic PD was diagnosed according to UK Parkinson’s disease Brain Bank Criteria. PD participants were assessed over three sessions (i) baseline, (ii) 18 months, and (iii) 36 months. PD exclusion criteria included; memory impairment (≤24 Mini Mental State Exam [MMSE]), dementia with Lewy bodies, drug-induced Parkinsonism, vascular Parkinsonism, atypical Parkinson’s syndromes, poor English language, and inability to consent. Participants were assessed “on” medication, defined as within 1 hour of medication intake.

To provide a comparison of cognitive decline with normal ageing, controls of a similar age and sex were recruited from community sources. Two control cohorts were recruited; the first cohort completed assessments at sessions 1 and 3, the second cohort completed assessments at all time points. Inclusion criteria included: >60 years of age; able to walk independently without an aid; absent of cognitive impairment (≤24 on MMSE) and absent of movement or mood disorders.

The study was approved by the Newcastle and North Tyneside Research and Ethics Committee.

### Clinical Assessment

Age, sex, height, weight, and depression (using Geriatric Depression Scale [GDS-15]) were recorded at each session. The National Adult Reading Test (NART) assessed premorbid intelligence at baseline. PD motor severity was assessed using the Movement Disorders Society Unified Parkinson’s Disease Rating Scale (MDS-UPDRS) Part III; Hoehn and Yahr Stage (H & Y); and levodopa equivalent daily dose (LEDD). Freezing of gait (FOG) was assessed with the FOG questionnaire. Comorbidities were self-reported by all participants.

### Gait Assessment

Participants walked for 2 minutes at a comfortable pace around a 25-m circuit inclusive of a 7 × 0.6m instrumented walkway (Platinum model GaitRite, CIR systems Inc, USA; Supplementary Figure 1). Gait assessment was completed under single task (ST) for which participants were asked to “concentrate on their walking” and dual task (DT) where participants were asked to “concentrate equally on their walking and a concurrent task”. The Wechsler Forward Digit Span was adopted as the concurrent task; a validated working memory task tailored to individual performance. Maximum digit span was first assessed in sitting, determined by longest span recalled in two of three attempts. Participants then recalled continuous strings of their maximum digit span while walking (13).

Gait outcomes were derived from a model of gait developed in older adults (14) and validated in PD (15). The model describes 16 discrete gait characteristics representing domains of pace, rhythm, variability, asymmetry, and postural control (Supplementary Figure 1).

### Cognitive Assessment

A comprehensive cognitive battery was completed at all sessions. Individual tests were represented by seven cognitive domains. *Global cognition:* measured using the MoCA. *Attention:* Cognitive Drug Research battery (CDR); simple reaction time (SRT), choice reaction time (CRT), and digit vigilance (DV). *Fluctuating attention (individual reaction time variability):* coefficient of variance (CV) of the SRT, CRT, and DV. *Visual memory*: Cambridge Neuropsychological Test Automated Battery (CANTAB); spatial recognition memory (SRM), pattern recognition memory (PRM), and paired associate learning (PAL). *Executive-function*: one touch stockings (OTS) from CANTAB, semantic fluency; naming animals in 90 seconds and the Hayling and Brixton (16). *Visuospatial function*: interlocking pentagon’s copying composite score from the MMSE. *Working memory* was assessed using Wechsler forward digit span (17). Additional references are available in Supplementary Material.

### Data Analysis

Analysis was conducted using SPSS (IBM Corp. V.21, USA) and R (R Foundation for Statistical Computing, V 3.2.2, Austria). The first stage was univariate to describe gait and cognitive data. Distribution of continuous variables was tested for normality using the Skewness–Kurtosis test and inspection of boxplots and histograms. Paired samples *t*-test examined differences in baseline and final assessment for clinical characteristics. Student’s *t*-test and chi-square test examined differences between completers and noncompleters (*p* = .05).

The second stage of analysis used linear mixed effects modelling (LMEM; R, “*lme4*”) to model cognitive decline and its predictors. Random intercept models gave each participant a unique intercept and regression coefficient. Initially, univariate analysis was conducted to determine cognitive assessments that significantly changed over time, those which changed significantly were entered into an adjusted model. The model was adjusted for covariates including baseline age, NART, and gender as fixed effects, as well as interactions of session with GDS-15 and LEDD. A backward stepwise method was employed to remove nonsignificant covariates.

Third, LMEM identified baseline gait characteristics predictors of cognitive decline in PD. Cognitive decline was determined for each domain using the strongest representative of each domain, i.e., greatest decline over time. Base models were constructed for each cognitive assessment (age, gender, NART, GDS-15, and LEDD), entered into the models as fixed effects. A backward stepwise method was employed. Gait characteristics under ST and DT at baseline were entered into the model as a fixed effect to determine whether gait characteristics in addition to covariates were a significant predictor of cognitive decline.

The final step identified whether baseline global cognition predicted change in cognition. MoCA and each gait characteristic at baseline were added to base models to assess which was a stronger predictor of cognitive decline. To further validate findings linear regression analysis was performed to identify whether baseline cognitive measures could predict change in the same measure. For analysis, cognitive change was the dependent variable with age, sex and NART entered in the first block and baseline cognition entered in the second block.

Log-likelihood ratio tests compared model fit. Analyses were conducted without adjustment for multiple comparisons. A stringent *p* value of ≤.01 guided interpretations.

## Results

### Study Participants

**Figure 1.**
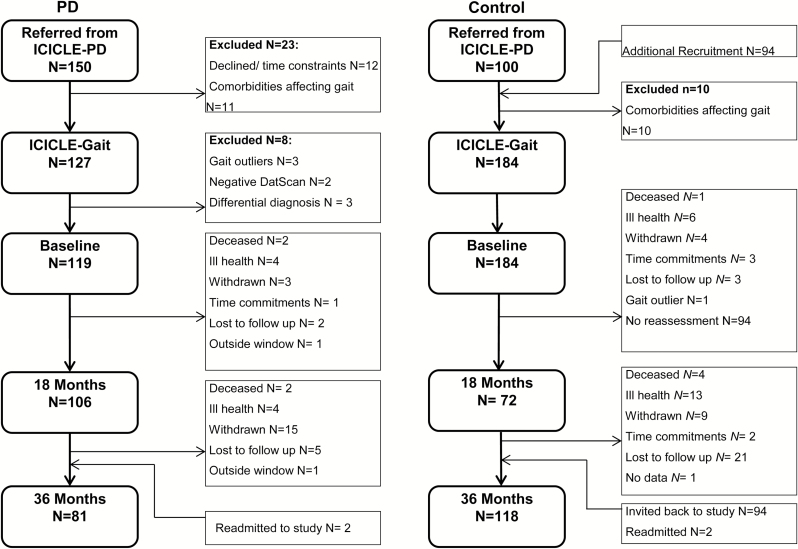
Flowchart of participants recruited and assessed throughout the ICICLE-Gait study.

Figure 1 summarizes participant recruitment and attrition in ICICE-Gait. Initially, 150 PD participants were referred, of whom 127 consented with 194 control subjects consented. After exclusions, 119 PD and 184 control subjects completed baseline assessment. At 18 months, 106 (89%) PD and 72 (39%) control participants completed assessments, at 36 months 81 (68%) PD and 118 (64%) control participants returned. Table 1 displays the clinical characteristics of participants at baseline and 36 months. The PD group contained proportionally more males throughout the study, whereas the control group contained proportionally more females. Baseline comorbidities in both groups were low and largely similar between groups (Supplementary Table 1). The average PD disease duration at baseline was 6.29 ± 4.67 months. Over 3 years, PD motor severity increased (*p* < .001) as did LEDD (*p* < .001). FOG significantly increased over 3 years (*p* < .001) but no participants experienced FOG during the gait assessment. Depression did not significantly change in either group. There were no significant differences in clinical demographics for PD participants who did and did not complete 36 month assessment (Supplementary Table 2).

**Table 1.** Demographic Characteristics, Mean (*SD*), of Participants at Baseline and 36 Months

Demographic	PD				Control			
	Baseline	36 Months	*T*	*p*	Baseline	36 Months	*T*	*p*
Sex (M and F)	79 and 40	55 and 26	—	—	78 and 106	53 and 64	—	—
Age (years)	66.11 (9.90)	69.13 (9.90)	−89.96	**<.01**	68.87 (7.10)	72.64 (7.06)	102.89	**<.01**
Height (m)	1.70 (.08)	1.69 (.09)	3.41	**<.01**	1.68 (0.10)	1.68 (0.09)	−2.39	**.02**
NART	115.02 (11.13)	—	—	—	117 (7.72)	—	—	—
Disease duration (months)	6.29 (4.67)	—	—	—	—	—	—	—
LEDD (mg/day)	172.26 (129.53)	515.05 (256.08)	−12.94	**<.01**	—	—	—	—
MDS-UPDRS III	24.97 (10.44)	38.04 (12.50)	−11.33	**<.01**	—	—	—	—
FOG	0.59 (2.54)	2.18 (5.33)	−2.71	**<.01**	—	—		
GDS	2.59 (2.23)	2.80 (2.41)	−0.89	.38	1.28 (2.03)	1.41 (2.34)	−0.71	.48
Hoehn and Yahr stage, *n* (%)	I (28)	I (1)	—	—	—	—	—	—
	II (70)	II (82)						
	III (21)	III (9)						
	IV (0)	IV (2)						

*Note:* FOG = freezing of gait; GDS = geriatric depression scale; LEDD = levodopa equivalent daily dose; MDS-UPDRS III = unified Parkinson’s disease rating scale; NART = national adult reading test; PD, Parkinson’s disease. Bold values represent significance level *p* ≤ 0.01.

### Baseline Gait

PD baseline gait characteristics can be found in Supplementary Table 3. Comparing gait characteristics at baseline between completers and noncompleters, those who withdrew had higher step length variability under ST and DT (*p* =·.02 and *p* ≤ .01, respectively) and higher step velocity variability under DT only (*p* ≤ .01). There were no other significant differences (Supplementary Table 4).

### Change in Cognition

Table 2 presents univariate and modelled change in cognition for both groups. Over 3 years, PD participants significantly declined on eight of 16 assessments. Attention declined on SRT (19.41 points per session [pps], *p* =·.01), CRT (36.65 pps, *p* < .01), and DV (8.89 pps, *p* < .01). Fluctuating attention declined on CRT CV (1.29 pps, *p* < .01). PD participants declined on executive function; OTS (0.75 pps, *p* < .01) and Brixton (0.31 pps, *p* < .01). Finally, PD participants declined on visual memory; SRM (0.71 pps, *p* < .01) and PAL (0.13 pps, *p* < .01). Control participants declined on two of 16 assessments; CRT (10.23 pps, *p* < .01) and SRM (0.37 pps, *p* < .01). Supplementary Table 5 presents all descriptive data for cognitive performance at each session. PD participants who withdrew before 36 months had worse baseline working memory, attention, fluctuating attention, and visual memory (Supplementary Table 6).

**Table 2.** Modelled Change in Cognitive Assessments Over 3 Years

	Univariate change				Modelled change adjusted				Univariate change				Modelled change adjusted			
	Change per session	*SE*	*T*	*p*	Change per session	*SE*	*T*	*p*	Change per session	*SE*	*T*	*p*	Change per session	*SE*	*T*	*p*
Global cognition																
MoCA	0.23	0.14	1.62	.12					**—**	**—**	**—**	**—**				
Working memory																
Forward digit span	0.03	0.06	0.50	.62					0.05	0.05	1.10	.27				
Attention																
Reaction time (mean)^2^	19.20	7.23	2.67	**.01**	19.41	7.31	2.66	**.01**	5.50	3.26	1.69	.09				
Choice reaction time (mean)^1,2^	37.02	5.77	6.42	**<.01**	36.65	5.76	6.36	**<.01**	9.97	3.46	2.88	**<.01**	10.23	3.46	2.96	**<.01**
Digit vigilance (mean)^1^	9.08	2.35	3.86	**<.01**	8.89	0.44	3.75	**<.01**	4.00	1.87	2.14	.03				
Fluctuating attention																
Reaction time (CV) (%)^3^	0.63	0.32	1.92	.06					0.28	0.30	0.95	.35				
Choice reaction time (CV) (%)^1^	1.31	0.29	4.47	**<.01**	1.29	0.29	4.45	**<.01**	0.50	0.21	2.43	.02				
Digit vigilance (CV) (%)^1^	0.57	0.26	2.17	.03					0.61	0.25	2.47	.02				
Executive function																
One touch stocking (prob. solved)^4^	−0.76	0.23	−3.35	**<.01**	−0.75	0.23	−3.34	**<.01**	−0.09	0.11	−0.83	.41				
Semantic fluency	−0.33	0.32	−1.03	.30					−0.66	0.31	−2.13	.04				
Hayling score^2^	0.04	0.09	0.43	.67					**—**	**—**	**—**	**—**				
Brixton score^2^	−0.33	0.12	−2.90	**<.01**	−0.31	0.11	−2.72	**<.01**	**—**	**—**	**—**	**—**				
Visual memory																
Pattern recognition memory (number correct)^2^	−0.20	0.13	−1.50	.14					−0.17	0.11	−1.50	.14				
Spatial recognition memory (number correct)^2^	−0.72	0.12	−5.89	**<.01**	−0.71	0.12	−5.83	**<.01**	−0.36	0.10	−3.62	**<.01**	−0.37	0.10	−3.76	**<.01**
Paired associate learning (mean trials to success)^2^	0.14	0.04	3.45	**<.01**	0.13	0.04	3.20	**<.01**	**—**	**—**	**—**	**—**				
Visuospatial																
Pentagons^2^	−0.06	0.03	−2.40	.02					0.02	0.02	1.21	.23				

*Note:* CV = coefficient of variance; MoCA = Montreal cognitive assessment; *SE* = standard error. Bold values represent significance level *p* ≤ 0.01.

Covariates; ^1^Age; ^2^Age, NART; ^3^Age, GDS; ^4^Age, NART, LEDD; ^5^Age, NART, GDS, sex.

### Gait Predicts Cognitive Decline at 36 Months

ST and DT predictors were similar in nature, thus only ST data is presented (DT available in Supplementary Table 7). Table 3 summarizes baseline ST gait predictors of cognitive decline. Decline in fluctuating attention was predicted by pace (slower velocity [*β* = 4.05, *p* < .01); reduced step length [*β* = 8.64, *p* < .01]), variability (increased swing time variability [*β* = 2.56, *p* < .01]; step stance variability [*β* = 1.98, *p* =·.01]; and step length variability [*β* = 115.68, *p* < .01]), and gait-related postural control (increased step width [*β* = 26.69, *p* < .01]). Visual memory decline was predicted by pace (reduced step length [*β* = 2.93, *p* =·01]). Prediction of decline in attention by variability was near significance (increased step length variability [*β* = 1639.29, *p* =·04]) but decline in executive function was not predicted by any gait characteristic. All gait characteristics improved the fit of the model except for step width as a predictor of fluctuating attention decline (*χ*^2^ = 5.91, *p* =·.05).

**Table 3.** LMEM Identifying Single Task Gait Characteristics and Global Cognition as Predictors of Cognitive Decline in PD

	Cognitive domain	Cognitive assessment	Predictor domain	Predictor	Regression coefficients			
					*β*	*SE*	*T*	*p*
Gait	Attention	CRT	Variability	Step length *SD* × session	1639.29	807.78	2.10	.04
	Fluctuating attention	CRTCV	Pace	Step velocity × session	−4.05	1.34	−3.02	**<.01**
				Step length × session	−8.64	2.86	−3.02	**<.01**
				Step swing *SD* × session	2.56	0.93	2.75	**<.01**
			Variability	Step time *SD* × session	2.14	0.90	2.38	.02
				Step stance *SD* × session	1.98	0.77	2.58	**.01**
				Step length *SD* × session	115.68	40.41	2.86	**<.01**
			Rhythm	Step stance time × session	0.01	0.03	2.26	.03
			Postural control	Step width × session	26.69	9.02	2.96	**<.01**
	Visual memory	SRM	Pace	Step velocity × session	1.32	0.56	2.33	.02
				Step length × session	2.93	1.19	2.47	**.01**
Cognition	Attention	CRT	Global cognition	MoCA × session	−5.68	1.54	−3.70	**<.01**
	Fluctuating attention	CRTCV			−1.68	0.80	−2.10	.04
	Visual memory	SRM			0.05	0.03	1.45	.15

*Note:* CRT = choice reaction time; CV = coefficient of variance; MoCA = Montreal cognitive assessment; *SD* = standard deviation; *SE* = standard error; SRM = spatial recognition memory. Bold values represent significance level *p* ≤ 0.01.

### Comparing Gait and Cognition as Predictors of Cognitive Decline

Compared to gait characteristics, baseline MoCA was a significant predictor of a decline in attention (*p* < .01) but could not predict decline in fluctuating attention (*p* = .04) or visual memory (*p* = .15; Table 3).

For decline in fluctuating attention, characteristics of pace (step velocity [*χ*^2^ = 10.93, *p* < .01], step length [*χ*^2^ = 11.22, *p* < .01]) and variability (step length variability [*χ*^2^ = 8.75, *p* = .01]) proved better predictors than MoCA. In addition, for visual memory decline pace (step velocity [*χ*^2^ = 8.70, *p* = .01] and step length [*χ*^2^ = 10.90, *p* < .01]) was a significant predictor over MoCA. However, other measures of pace (swing time variability [*χ*^2^ = 6.73, *p* = .03]) and variability (stance time variability [*χ*^2^ = 7.11, *p* = .03]) did not improve model fit for decline in fluctuating attention, although these but not MoCA remained significant in the model. Additional linear regression analysis revealed baseline CRTCV was unable to predict CRTCV decline (*p* = .75; Supplementary Table 8).

## Discussion

To our knowledge, this is the first study to demonstrate that gait is able to predict cognitive decline in early PD. Moreover, this was a large, incident cohort study followed from diagnosis allowing for prognostic significance of gait in early disease to be determined. Gait predicted decline in specific cognitive domains (fluctuating attention and visual memory) over 3 years which was selective to discrete gait characteristics. Importantly, gait was a stronger predictor than baseline cognition. We therefore provide the first evidence for the utility of gait as a noninvasive clinical biomarker for early cognitive decline in PD.

### Gait Predicts Cognitive Decline

Cognitive decline in fluctuating attention and visual memory was independently predicted by gait characteristics represented by domains of pace, variability, and postural control (15). Slower pace, higher gait variability, and more unstable postural control at diagnosis predicted decline in fluctuating attention. Additionally, slower pace predicted decline in visual memory. By comparison, characteristics representing rhythm and asymmetry were unable to predict cognitive decline. Related evidence comes from robust associations between gait and cognition in cross-sectional studies in early PD (10) which supports our findings. Parallels can be drawn from studies in older adults showing prognostic associations between slow gait speed and increased gait variability with cognitive decline (6,7). In advanced PD, gait impairment on the MDS-UPDRS III predicts future PDD (11) further demonstrating the sensitivity of gait to cognitive decline. However, in very early disease, tools with increased sensitivity are necessary to provide effective clinical biomarkers and derive novel therapeutics. Thus, highlighting the need for quantitative gait analysis. Importantly, comprehensive gait analysis can be assessed in clinical practice with current technological advances improving future feasibility.

Progression of cognitive impairment over 36 months in our cohort was characterized by attention, fluctuating attention, executive-function, and visual memory similar to other incident cohorts (18,19). Muslimovic *et al*. observed greatest decline in attention and psychomotor speed (19). The Campaign study found visual memory and executive-function were early cognitive features to decline (18). Notably, this group did not assess attention; therefore, we cannot draw parallels with this domain.

### Is Fluctuating Attention a Marker of Dementia?

We were able to predict change in fluctuating attention and visual memory, which is highly relevant given their contribution to cognitive decline evolution and dementia in PD. Fluctuating attention was particularly sensitive to baseline gait. Previous work shows these cognitive features may be important precursors for PDD. For example, fluctuating attention (measured by CRT SD) is significantly worse in Lewy body dementias (an overarching term comprising both PDD and DLB) compared to Alzheimer's disease (AD) (20). Critically, variability on CRT most strongly reflects fluctuating cognition in DLB (21). Visual and memory components play a vital role in SRM which is temporally mediated; however, lesion (22) and cognitive cohort studies (23) suggest frontal involvement, indicating overlap of underlying mechanisms.

### Potential Underlying Mechanisms

Underlying pathology of gait and cognition is poorly understood but evidence suggests they share common substrates. Gait is not purely dopaminergic (24) and interacts with other neurotransmitter systems. Previous work implicates the cholinergic system in gait dysfunction, demonstrated by short-latency afferent inhibition (25) and intervention (26) studies. The cholinergic system also has an essential role in attention (27) likely to stem from nucleus basalis of Meynert (nbM) (27,28), which may also mediate visual deficits (28). Additionally, the role of amyloid pathology in early gait impairment in PD (29) demonstrates a combination of pathological substrates of gait and cognitive decline.

Interestingly, gait was unable to predict executive-function decline, an early feature of cognitive decline (18). This may reflect the overarching role of attention in mediating cognitive function (30), or sensitivity of attentional over executive-function measures in early disease.

### Comparison of Gait and Cognitive Outcomes

We were also interested to see if discrete gait characteristics were more sensitive than cognitive measures to early cognitive decline. Our findings suggest that this is the case. Global cognition did not predict decline in fluctuating attention or visual memory. Furthermore, additional analysis showed that fluctuating attention did not predict future cognition decline. These findings strengthen the case for the role of discrete gait characteristics as predictors of cognitive decline in early PD.

We extended knowledge of gait as a prognostic marker by taking a comprehensive measurement approach which highlighted the specificity of gait to cognitive decline. We also evaluated gait under DT because it sensitizes the relationship between cognition and gait and is frequently incorporated into protocols. In contrast to earlier work (31), our DT cognitive associations were not significantly different from older adults, partly reflecting our DT paradigm which controls for baseline cognitive capacity (13).

## Limitations

This study has several limitations. First, although our cognitive battery was comprehensive for attention, executive function, and memory, we acknowledge that not all aspects of these domains were assessed. Our cognitive battery was also less comprehensive for visuospatial function. Significant visuospatial decline was not apparent in our cohort but other work identified pentagons decline in early PD (18) suggesting this assessment was adequate. Second, the longitudinal nature of the study inevitably leads to attrition. Attrition rates totaled 32%, comparable to similarly designed studies (18,19). Baseline scores revealed that those who withdrew were worse on cognitive assessments (Supplementary Table 3). This may indicate those with more rapid decline were more likely to withdraw and would have been of interest to this study. In an attempt to alleviate bias, LMEM were chosen as this technique is able to handle missing data yet it is possible rate of cognitive decline was underestimated. Thirdly, the population was drawn from an incident PD cohort followed from diagnosis with repeat assessments every 18 months. Although misdiagnosis may have contributed, this is unlikely to have made a major impact. Diagnosis followed a stringent process, applying the Queen’s Square Brain Bank Criteria at each assessment, and revised diagnosis revealed that the numbers were low. In addition, the bidirectional relationship between gait and cognition should be acknowledged. A number of studies have explored this relationship in older adults (32) but not in PD (10); however, this was beyond the scope of the present study. Finally, our findings need to be replicated in an independent cohort.

## Conclusions

This is the first study to identify gait as a predictor of cognitive decline in a large incident PD cohort. Furthermore, it was identified that gait is sensitive to decline in specific cognitive domains in PD. Our work focused on specific assessments of cognitive decline, a critical approach providing further understanding of the underlying pathology of gait and cognition. Future work will focus on gait as a predictor of PDD as the cohort evolves.

## Supplementary Material

Supplementary data is available at *The Journals of Gerontology, Series A: Biological Sciences and Medical Sciences* online.

## Funding

This work was supported by Parkinson’s UK (J-0802, G-1301) and the National Institute for Health Research (NIHR) Biomedical Research Unit based at Newcastle upon Tyne Hospitals NHS Foundation Trust and Newcastle University. This work was also supported by the NIHR Newcastle Biomedical Research Centre and Newcastle CRF Infrastructure funding.

## Supplementary Material

Supplementary_Table_1

Supplementary_Table_2

Supplementary_Table_3

Supplementary_Table_4

Supplementary_Table_5

Supplementary_Table_6

Supplementary_Table_7

Supplementary_Table_8

Supplementary_References
