# Supplementary material for: Gait Rather Than Cognition Predicts Decline in Specific Cognitive Domains in Early Parkinson’s Disease
Source: J Gerontol A Biol Sci Med Sci. 2017 May 3;72(12):1656–62. doi: 10.1093/gerona/glx071 (PMC5861960; doi:10.1093/gerona/glx071)
Supplement: Supplementary_Table_1 [file glx071_suppl_supplementary_table_1.docx]

| **Comorbidity** | **PD**  **n (%)** | **Control**  **n (%)** | **Chi-squared** |
| --- | --- | --- | --- |
| Ischemic heart disease | 11 (9%) | 8 (4%) | 2.95 |
| Diabetes Mellitus I | 9 (8%) | 4 (2%) | **5.11*** |
| Diabetes Mellitus II | 7 (6%) | 6 (3%) | 1.21 |
| Hypertension | 38 (32%) | 48 (26%) | 1.22 |
| Hypercholesterolemia | 15 (13%) | 24 (13%) | 0.01 |
| Stroke/TIA | 9 (8%) | 1 (1%) | **11.16**** |

**Supplementary Table 1**. Baseline comorbidities in all participants.

*Chi-squared test; *p≤0.05, ** p≤0.01.*
