# Supplementary material for: Gait Rather Than Cognition Predicts Decline in Specific Cognitive Domains in Early Parkinson’s Disease
Source: J Gerontol A Biol Sci Med Sci. 2017 May 3;72(12):1656–62. doi: 10.1093/gerona/glx071 (PMC5861960; doi:10.1093/gerona/glx071)
Supplement: Supplementary_Table_2 [file glx071_suppl_supplementary_table_2.docx]

**Supplementary Table 2.** Demographic data for PD participants who did and did not complete assessments at 36 months.

| **Demographic** |  | **Completers (*n*=81)** | |  | **Non-completers (*n*=38)** | |  | **T** | ***p*** |
| --- | --- | --- | --- | --- | --- | --- | --- | --- | --- |
|  |  | *Mean* | *SD* |  | *Mean* | *SD* |  |  |  |
| **Sex (M & F)** |  | 55M & 26F | |  | 24M & 14F | |  | .26* | .68* |
| **Age (years)** |  | 66.11 | 9.91 |  | 68.72 | 11.60 |  | -1.27 | .21 |
| **Height (m)** |  | 1.70 | .08 |  | 1.69 | .08 |  | .93 | .35 |
| **NART** |  | 115.26 | 11.08 |  | 114.49 | 11.37 |  | .35 | .73 |
| **LEDD (mg/day)** |  | 158.86 | 114.67 |  | 212.20 | 188.31 |  | -1.61 | .11 |
| **UPDRS III** |  | 24.35 | 10.32 |  | 27.55 | 10.37 |  | -1.58 | .12 |
| **FOG** |  | 0.58 | 2.53 |  | 0.89 | 3.11 |  | -.59 | .26 |
| **GDS** |  | 2.65 | 2.30 |  | 2.50 | 1.89 |  | .36 | .72 |
| **Hoehn & Yahr stage n (%)** |  | I (21) II (47) III (13) IV (0) | |  | I (7) II (23) III (8) IV (0) | |  | - | |

*NART, national adult reading test; LEDD, levodopa equivalent daily dose; UPDRS III, unified Parkinson’s disease rating scale; FOG, freezing of gait questionnaire; GDS, geriatric depression scale. All student’s t-test apart from sex (Chi-square*).*
