# Supplementary material for: Gait Rather Than Cognition Predicts Decline in Specific Cognitive Domains in Early Parkinson’s Disease
Source: J Gerontol A Biol Sci Med Sci. 2017 May 3;72(12):1656–62. doi: 10.1093/gerona/glx071 (PMC5861960; doi:10.1093/gerona/glx071)
Supplement: Supplementary_Table_3 [file glx071_suppl_supplementary_table_3.docx]

**Supplementary Table 3**. Gait characteristics in PD at baseline.

| **Gait Domain** | **Gait Variable** |  | **Single Task** | |  | **Dual Task** | |
| --- | --- | --- | --- | --- | --- | --- | --- |
|  |  |  | *Mean* | *SD* |  | *Mean* | *SD* |
| **Pace** |  |  |  |  |  |  |  |
|  | Step velocity (*m/s*) |  | 1.12 | 0.21 |  | 1.06 | 0.22 |
|  | Step Length (*m*) |  | 0.62 | 0.10 |  | 0.59 | 0.10 |
|  | Swing time SD (*ms*) |  | 2.81 | 0.32 |  | 2.92 | 0.32 |
| **Variability** |  |  |  |  |  |  |  |
|  | Step time SD (*ms*) |  | 2.88 | 0.33 |  | 3.04 | 0.36 |
|  | Stance time SD (*ms*) |  | 3.06 | 0.38 |  | 3.27 | 0.41 |
|  | Step velocity SD (*m/s*) |  | 0.054 | 0.017 |  | 0.060 | 0.018 |
|  | Step length SD (*m*) |  | 0.023 | 0.009 |  | 0.025 | 0.009 |
| **Rhythm** |  |  |  |  |  |  |  |
|  | Step time (*ms*) |  | 559.89 | 48.74 |  | 571.05 | 53.41 |
|  | Swing time (*ms*) |  | 391.83 | 33.20 |  | 391.58 | 34.84 |
|  | Stance time (*ms*) |  | 728.40 | 76.80 |  | 751.03 | 85.45 |
| **Asymmetry** |  |  |  |  |  |  |  |
|  | Step time asymmetry (*ms*) |  | 4.15 | 2.34 |  | 4.51 | 2.52 |
|  | Swing time asymmetry (*ms*) |  | 3.69 | 1.97 |  | 3.98 | 1.97 |
|  | Stance time asymmetry (*ms*) |  | 3.67 | 1.93 |  | 3.95 | 2.01 |
| **Postural Control** |  |  |  |  |  |  |  |
|  | Step length asymmetry (*m*) |  | 0.146 | 0.067 |  | 0.149 | 0.076 |
|  | Step width (*m*) |  | 0.093 | 0.031 |  | 0.095 | 0.032 |
|  | Step with SD (*m*) |  | 0.019 | 0.006 |  | 0.018 | 0.005 |
