# Supplementary material for: Gait Rather Than Cognition Predicts Decline in Specific Cognitive Domains in Early Parkinson’s Disease
Source: J Gerontol A Biol Sci Med Sci. 2017 May 3;72(12):1656–62. doi: 10.1093/gerona/glx071 (PMC5861960; doi:10.1093/gerona/glx071)
Supplement: Supplementary_Table_4 [file glx071_suppl_supplementary_table_4.docx]

|  |  |  | | **Single Task** | |  | |  |  | | **Dual Task** | |  | |
| --- | --- | --- | --- | --- | --- | --- | --- | --- | --- | --- | --- | --- | --- | --- |
| **Gait Domain** | **Gait Variable** | **Completers (*n*=81)** | | **Non-Completers (*n*=38)** | | **T-Test** | |  | **Completers (*n*=80)** | | **Non-Completers (*n*=39)** | | **T-Test** | |
|  |  |  |  |  |  |  |  |  |  |  |  |  |  |  |
|  |  | *Mean* | *SD* | *Mean* | *SD* | *T* | *p* |  | *Mean* | *SD* | *Mean* | *SD* | *T* | *p* |
| **Pace** |  |  |  |  |  |  |  |  |  |  |  |  |  |  |
|  | Step velocity (*m/s*) | 1.14 | 0.21 | 1.08 | 0.22 | 1.41 | 0.16 |  | 1.08 | 0.23 | 1.02 | 0.20 | 1.44 | 0.15 |
|  | Step Length (*m*) | 0.63 | 0.10 | 0.60 | 0.11 | 1.55 | 0.12 |  | 0.61 | 0.10 | 0.57 | 0.10 | 1.73 | 0.09 |
|  | Swing time SD (*ms*) | 2.79 | .28 | 2.88 | 0.39 | -1.44 | 0.15 |  | 2.89 | 0.31 | 2.96 | 0.35 | -1.12 | 0.27 |
| **Variability** |  |  |  |  |  |  |  |  |  |  |  |  |  |  |
|  | Step time SD (*ms*) | 2.85 | 0.30 | 2.95 | 0.38 | -1.56 | 0.12 |  | 3.00 | 0.34 | 3.13 | 0.37 | -1.94 | 0.06 |
|  | Stance time SD (*ms*) | 3.02 | 0.36 | 3.16 | 0.43 | -1.97 | 0.05 |  | 3.22 | 0.40 | 3.38 | 0.43 | -2.00 | 0.05 |
|  | Step velocity SD (*ms*) | 0.052 | 0.014 | 0.057 | 0.213 | -1.42 | 0.16 |  | 0.057 | 0.016 | 0.066 | 0.018 | -2.95 | **<.01** |
|  | Step length SD (*m*) | 0.022 | 0.006 | 0.025 | 0.010 | -2.29 | **0.02** |  | 0.023 | 0.008 | 0.029 | 0.009 | **-**3.15 | **<.01** |
| **Rhythm** |  |  |  |  |  |  |  |  |  |  |  |  |  |  |
|  | Step time (*ms*) | 558.84 | 45.47 | 562.15 | 55.66 | -.034 | 0.73 |  | 571.20 | 52.07 | 570.74 | 56.74 | .044 | 0.97 |
|  | Swing time (*ms*) | 392.25 | 33.19 | 390.94 | 33.63 | 0.20 | 0.84 |  | 393.48 | 35.01 | 387.67 | 34.60 | 0.85 | 0.40 |
|  | Stance time (*ms*) | 725.76 | 72.43 | 734.01 | 86.10 | -0.54 | 0.59 |  | 749.36 | 84.82 | 754.43 | 87.73 | -0.30 | 0.76 |
| **Asymmetry** |  |  |  |  |  |  |  |  |  |  |  |  |  |  |
|  | Step time asymmetry (*ms*) | 4.13 | 2.34 | 4.21 | 2.36 | -0.18 | 0.86 |  | 4.58 | 2.48 | 4.36 | 2.62 | 0.44 | 0.66 |
|  | Swing time asymmetry (*ms*) | 3.73 | 2.01 | 3.61 | 1.89 | 0.32 | 0.75 |  | 4.10 | 1.98 | 3.75 | 1.95 | 0.89 | 0.38 |
|  | Stance time asymmetry (*ms*) | 3.70 | 1.99 | 3.61 | 1.82 | 0.24 | 0.81 |  | 4.01 | 2.07 | 3.84 | 1.89 | 0.44 | 0.66 |
| **Postural Control** |  |  |  |  |  |  |  |  |  |  |  |  |  |  |
|  | Step length asymmetry (*m*) | 0.141 | 0.071 | 0.154 | 0.057 | -0.97 | 0.33 |  | 0.148 | 0.075 | 0.150 | 0.079 | -0.14 | 0.89 |
|  | Step width (*m*) | 0.091 | 0.032 | 0.095 | 0.027 | -0.60 | 0.55 |  | 0.094 | 0.033 | 0.097 | 0.030 | -0.55 | 0.58 |
|  | Step with SD (*m*) | 0.019 | 0.006 | 0.019 | 0.005 | -0.24 | 0.81 |  | 0.018 | 0.005 | 0.019 | 0.005 | -0.83 | 0.41 |

**Supplementary Table 4.** Baseline gait characteristics for PD participants who did and did not complete 36 month assessment.
