# Supplementary material for: Gait Rather Than Cognition Predicts Decline in Specific Cognitive Domains in Early Parkinson’s Disease
Source: J Gerontol A Biol Sci Med Sci. 2017 May 3;72(12):1656–62. doi: 10.1093/gerona/glx071 (PMC5861960; doi:10.1093/gerona/glx071)
Supplement: Supplementary_Table_5 [file glx071_suppl_supplementary_table_5.docx]

**Supplementary Table 5.** Descriptive data of cognitive assessments at baseline, 18 and 36 months.

|  | **PD** | | | | | | | |  | **Control** | | | | | | | |
| --- | --- | --- | --- | --- | --- | --- | --- | --- | --- | --- | --- | --- | --- | --- | --- | --- | --- |
|  | **BL** | |  | **18 Months** | |  | **36 Months** | |  | **BL** | |  | **18 Months** | |  | **36 Months** | |
|  | *Mean* | *SD* |  | *Mean* | *SD* |  | *Mean* | *SD* |  | *Mean* | *SD* |  | *Mean* | *SD* |  | *Mean* | *SD* |
| **Global Cognition** |  |  |  |  |  |  |  |  |  |  |  |  |  |  |  |  |  |
| MoCA | 25.22 | 3.57 |  | 26.05 | 3.62 |  | 25.74 | 4.03 |  | - | - |  | - | - |  | - | - |
| **Working memory** |  |  |  |  |  |  |  |  |  |  |  |  |  |  |  |  |  |
| Forward digit span | 5.82 | 1.11 |  | 5.97 | 1.38 |  | 5.94 | 1.17 |  | 6.18 | 1.22 |  | 6.19 | 1.25 |  | 6.30 | 1.13 |
| **Attention** |  |  |  |  |  |  |  |  |  |  |  |  |  |  |  |  |  |
| Reaction time (mean) | 347.48 | 101.16 |  | 361.5 | 90.95 |  | 387.10 | 141.28 |  | 325.03 | 63.46 |  | 316.59 | 61.07 |  | 331.34 | 74.69 |
| Choice reaction time (mean) | 528.95 | 85.64 |  | 569.92 | 117.06 |  | 598.01 | 162.79 |  | 521.75 | 74.13 |  | 525.47 | 77.93 |  | 540.08 | 82.95 |
| Digit Vigilance (mean) | 479.53 | 56.27 |  | 483.23 | 63.8 |  | 496.19 | 65.30 |  | 455.33 | 49.94 |  | 458.15 | 47.92 |  | 461.44 | 45.75 |
| **Fluctuating Attention** |  |  |  |  |  |  |  |  |  |  |  |  |  |  |  |  |  |
| Reaction time (CV) (%) | 17.00 | 5.55 |  | 17.88 | 6.41 |  | 18.21 | 6.13 |  | 17.19 | 5.64 |  | 17.86 | 5.01 |  | 17.69 | 5.73 |
| Choice reaction time (CV) (%) | 18.93 | 3.85 |  | 20.62 | 6.00 |  | 21.18 | 6.21 |  | 17.88 | 3.90 |  | 18.38 | 4.50 |  | 18.86 | 4.43 |
| Digit Vigilance (CV) (%) | 16.08 | 3.74 |  | 16.35 | 4.36 |  | 17.17 | 4.95 |  | 14.65 | 3.91 |  | 14.94 | 4.22 |  | 15.83 | 5.51 |
| **Executive Function** |  |  |  |  |  |  |  |  |  |  |  |  |  |  |  |  |  |
| One touch stocking (problems solved) | 14.06 | 4.30 |  | 14.49 | 5.00 |  | 12.70 | 5.91 |  | 15.92 | 3.15 |  | 16.89 | 2.11 |  | 15.82 | 3.26 |
| Semantic Fluency (animals in 90 secs) | 21.77 | 6.38 |  | 22.14 | 7.12 |  | 21.34 | 8.12 |  | 24.24 | 6.06 |  | 24.18 | 6.39 |  | 23.12 | 5.18 |
| Hayling Score | 5.28 | 1.68 |  | 5.43 | 1.62 |  | 5.43 | 1.64 |  | - | - |  | - | - |  | - | - |
| Brixton Score | 4.54 | 2.36 |  | 3.99 | 2.48 |  | 4.04 | 2.47 |  | - | - |  | - | - |  | - | - |
| **Visual Memory** |  |  |  |  |  |  |  |  |  |  |  |  |  |  |  |  |  |
| Pattern Recognition memory (number correct) | 19.91 | 2.78 |  | 19.96 | 2.97 |  | 19.66 | 3.52 |  | 20.81 | 2.30 |  | 20.75 | 2.69 |  | 20.71 | 2.65 |
| Spatial Recognition memory (number correct) | 15.46 | 2.18 |  | 14.58 | 2.75 |  | 14.11 | 2.35 |  | 16.20 | 1.86 |  | 15.71 | 2.06 |  | 15.58 | 2.17 |
| Paired associate learning (mean trials to success) | 2.10 | 0.85 |  | 2.26 | 0.93 |  | 2.27 | 1.11 |  | - | - |  | - | - |  | - | - |
| **Visuospatial** |  |  |  |  |  |  |  |  |  |  |  |  |  |  |  |  |  |
| Pentagon copying | 1.91 | 0.29 |  | 1.74 | 0.57 |  | 1.81 | 0.52 |  | 1.91 | 0.31 |  | 1.95 | 0.22 |  | 1.94 | 0.27 |
