# Supplementary material for: Gait Rather Than Cognition Predicts Decline in Specific Cognitive Domains in Early Parkinson’s Disease
Source: J Gerontol A Biol Sci Med Sci. 2017 May 3;72(12):1656–62. doi: 10.1093/gerona/glx071 (PMC5861960; doi:10.1093/gerona/glx071)
Supplement: Supplementary_Table_6 [file glx071_suppl_supplementary_table_6.docx]

|  | **Completers (n=81)** | | **Non-Completers (n=38)** | | **T-test (BL-36)** | |
| --- | --- | --- | --- | --- | --- | --- |
|  | *Mean* | *SD* | *Mean* | *SD* | *T* | *P* |
| **Global Cognition** |  |  |  |  |  |  |
| MMSE | 28.83 | 1.09 | 28.32 | 1.63 | 1.76 | .08 |
| MoCA | 25.64 | 3.38 | 24.35 | 3.83 | 1.83 | .07 |
| **Working memory** |  |  |  |  |  |  |
| Forward digit span | 5.98 | 1.14 | 5.47 | 0.98 | 2.34 | **.02** |
| **Attention** |  |  |  |  |  |  |
| Reaction time (mean) | 343.71 | 114.25 | 355.31 | 66.86 | -0.58 | .56 |
| Choice reaction time (mean) | 520.02 | 79.37 | 547.51 | 95.84 | -1.64 | .10 |
| Digit Vigilance (mean) | 471.11 | 52.19 | 497.04 | 61.00 | -2.38 | **.02** |
| **Fluctuating Attention** |  |  |  |  |  |  |
| Reaction time (CV) (%) | 16.51 | 5.69 | 18.01 | 5.16 | -1.38 | .17 |
| Choice reaction time (CV) (%) | 18.39 | 3.34 | 20.05 | 4.58 | -1.99 | **.05** |
| Digit Vigilance (CV) (%) | 15.84 | 3.52 | 16.57 | 4.16 | -0.93 | .33 |
| **Executive Function** |  |  |  |  |  |  |
| One touch stocking (problems solved) | 14.35 | 3.65 | 13.44 | 5.44 | 1.05 | .39 |
| Semantic Fluency (animals in 90 secs) | 22.56 | 6.05 | 20.08 | 6.82 | 1.97 | .05 |
| Hayling Score | 5.44 | 1.61 | 4.92 | 1.81 | 1.58 | .12 |
| Brixton Score | 4.53 | 2.36 | 4.56 | 2.41 | -0.05 | .96 |
| **Visual Memory** |  |  |  |  |  |  |
| Pattern Recognition memory (number correct) | 20.14 | 2.86 | 19.19 | 2.87 | 1.64 | .10 |
| Spatial Recognition memory (number correct) | 15.73 | 2.16 | 14.75 | 2.32 | 2.19 | **.03** |
| Paired associate learning (mean trials to success) | 1.95 | 0.67 | 2.54 | 1.32 | -3.13 | **.02** |
| **Visuospatial** |  |  |  |  |  |  |
| Pentagon copying | 1.94 | 0.24 | 1.84 | 0.37 | 1.50 | .14 |

**Supplementary Table 6.** Cognitive data for PD participants who did and did not complete 36 month assessments.
