# Supplementary material for: Gait Rather Than Cognition Predicts Decline in Specific Cognitive Domains in Early Parkinson’s Disease
Source: J Gerontol A Biol Sci Med Sci. 2017 May 3;72(12):1656–62. doi: 10.1093/gerona/glx071 (PMC5861960; doi:10.1093/gerona/glx071)
Supplement: Supplementary_Table_7 [file glx071_suppl_supplementary_table_7.docx]

**Supplementary Table 7**. Linear mixed effects models identifying significant gait characteristics as predictors of cognitive decline under dual task conditions in PD.

| **Cognitive Domain** | **Cognitive Assessment** | **Predictor Domain** | **Predictor Characteristic** | **Regression Coefficients** | | | |
| --- | --- | --- | --- | --- | --- | --- | --- |
|  |  |  |  | *β* | *SE* | *T* | *P* |
| Attention | CRT | *Variability* | Step Time SD x Session | 42.54 | 16.30 | 2.61 | **0.01** |
| Fluctuating Attention | CRTCV | *Pace* | Step Velocity x Session | -3.86 | 1.24 | -3.11 | **<0.01** |
| Fluctuating Attention | CRTCV | *Pace* | Step Length x Session | -8.37 | 2.69 | -3.12 | **<0.01** |
| Fluctuating Attention | CRTCV | *Pace* | Step Swing SD x Session | 2.83 | 0.89 | 3.17 | **<0.01** |
| Fluctuating Attention | CRTCV | *Variability* | Step Time SD x Session | 2.35 | 0.82 | 2.87 | **0.01** |
| Fluctuating Attention | CRTCV | *Rhythm* | Step Stance time x Session | 0.01 | 0.003 | 2.93 | **<0.01** |
| Fluctuating Attention | CRTCV | *Asymmetry* | Stance Asymmetry x Session | 0.36 | 0.14 | 2.55 | **0.01** |
| Fluctuating Attention | CRTCV | *Postural control* | Step Width x Session | 27.70 | 8.86 | 3.13 | **<0.01** |
| Visual Memory | SRM | *Pace* | Step Velocity x Session | 1.36 | 0.52 | 2.60 | **0.01** |
| Visual Memory | SRM | *Pace* | Step Length x Session | 3.00 | 1.13 | 2.65 | **<0.01** |
| Visual Memory | SRM | *Variability* | Step Stance SD x Session | -0.76 | 0.30 | -2.51 | **0.01** |
