# Supplementary material for: Gait Rather Than Cognition Predicts Decline in Specific Cognitive Domains in Early Parkinson’s Disease
Source: J Gerontol A Biol Sci Med Sci. 2017 May 3;72(12):1656–62. doi: 10.1093/gerona/glx071 (PMC5861960; doi:10.1093/gerona/glx071)
Supplement: Supplementary_Table_8 [file glx071_suppl_supplementary_table_8.docx]

**Supplementary Table 8**. Multivariate linear regression model signifying predictors of change in fluctuating attention over three years in PD.

| **Dependent Variable** |  | **Predictors** | **β** | **P** | **R** | **Adjusted R^2^** | **F Change** | **ANOVA Sig** |
| --- | --- | --- | --- | --- | --- | --- | --- | --- |
| *Fluctuating Attention (CRTCV)* | Model 1 | Age | .151 | .162 | .236 | .022 | 1.648 | .185 |
|  |  | NART | -.065 | .541 |  |  |  |  |
|  |  | Gender | -.152 | .160 |  |  |  |  |
|  |  |  |  |  |  |  |  |  |
|  | Model 2 | Age | .156 | .155 | .238 | .011 | .102 | .297 |
|  |  | NART | -.068 | .526 |  |  |  |  |
|  |  | Gender | -.156 | .154 |  |  |  |  |
|  |  | CRT CV BL | -.035 | .750 |  |  |  |  |

*NART= national adult reading test, CRT CV= choice reaction time coefficient of variance, BL= baseline. CRTCV change score was entered as the dependent variable with age, sex and NART entered in the first block and baseline CRTCV entered in the second block as independent variables.*
