# Supplementary material for: Gait Rather Than Cognition Predicts Decline in Specific Cognitive Domains in Early Parkinson’s Disease
Source: J Gerontol A Biol Sci Med Sci. 2017 May 3;72(12):1656–62. doi: 10.1093/gerona/glx071 (PMC5861960; doi:10.1093/gerona/glx071)
Supplement: Supplementary_References [file glx071_suppl_supplementary_references.docx]

**Supplementary Methodological References:**

UK Parkinson’s Brain Bank Criteria(1)

MMSE (2)

MoCA (3)

Semantic Fluency (4)

CDR (5)

CANTAB (6)

Pentagons (7)

NART(8)

GDS(9)

MDS-UPDRS(10)

Hoehn and Yahr(11)

Levodopa equivalent daily dose(12)

R Programme(13)

Lmem 4 package (14)

1. Hughes AJ, Daniel SE, Kilford L, Lees AJ. Accuracy of clinical diagnosis of idiopathic Parkinson's disease: a clinico-pathological study of 100 cases. Journal of neurology, neurosurgery, and psychiatry. 1992;**55**:181-184.

2. Folstein MF, Folstein SE, McHugh PR. “Mini-mental state”: A practical method for grading the cognitive state of patients for the clinician. Journal of Psychiatric Research. 1975;**12**:189-198.

3. Nasreddine ZS, Phillips NA, Bédirian V, Charbonneau S, Whitehead V, Collin I*, et al.* The Montreal Cognitive Assessment, MoCA: a brief screening tool for mild cognitive impairment. Journal of the American Geriatrics Society. 2005;**53**:695-699.

4. Goodglass H, Kaplan E, Barresi B. The assessment of aphasia and related disorders. Lippincott Williams & Wilkins; 2001.

5. Nicholl CG, Lynch S, Kelly CA, White L, Simpson PM, Wesnes KA*, et al.* The cognitive drug research computerized assessment system in the evaluation of early dementia-is speed of the essence? International Journal of Geriatric Psychiatry. 1995;**10**:199-206.

6. Robbins TW, James M, Owen AM, Sahakian BJ, McInnes L, Rabbitt P. Cambridge Neuropsychological Test Automated Battery (CANTAB): A Factor Analytic Study of a Large Sample of Normal Elderly Volunteers. Dementia and Geriatric Cognitive Disorders. 1994;**5**:266-281.

7. Ala TA, Hughes LF, Kyrouac GA, Ghobrial MW, Elble RJ. Pentagon copying is more impaired in dementia with Lewy bodies than in Alzheimer's disease. Journal of Neurology, Neurosurgery & Psychiatry. 2001;**70**:483-488.

8. Nelson HE, O'Connell A. Dementia: The Estimation of Premorbid Intelligence Levels Using the New Adult Reading Test. Cortex. 1978;**14**:234-244.

9. Yesavage JA, Brink TL, Rose TL, Lum O, Huang V, Adey M*, et al.* Development and validation of a geriatric depression screening scale: A preliminary report. Journal of Psychiatric Research. 1982;**17**:37-49.

10. Goetz CG, Tilley BC, Shaftman SR, Stebbins GT, Fahn S, Martinez-Martin P*, et al.* Movement Disorder Society-sponsored revision of the Unified Parkinson's Disease Rating Scale (MDS-UPDRS): Scale presentation and clinimetric testing results. Movement Disorders. 2008;**23**:2129-2170.

11. Hoehn MM, Yahr MD. Parkinsonism: Onset, progression and mortality. Neurology. 2001;**57**:S11-S26.

12. Tomlinson CL, Stowe R, Patel S, Rick C, Gray R, Clarke CE. Systematic review of levodopa dose equivalency reporting in Parkinson's disease. Movement Disorders. 2010;**25**:2649-2653.

13. R Core Team. R: a language and environment for statistical computing. Vienna: R Foundation for Statistical Computing; 2013.

14. Bates D MM, Bolker B and Walker S. lme4: Linear mixed effects models using Eigen and S4. Journal of Statistical Software. 2014.
